# Genicular nerve radiofrequency ablation: a systematic review of application for perioperative pain control in total knee arthroplasty and as treatment for chronic pain in well-appearing total knee arthroplasty

**DOI:** 10.1186/s43019-024-00222-9

**Published:** 2024-05-19

**Authors:** Chidebelum O. Nnake, Mouhanad M. El-Othmani, H. John Cooper, Roshan P. Shah, Jeffrey A. Geller, Alexander L. Neuwirth

**Affiliations:** Division of Hip and Knee Reconstruction, Columba University Medical Center, New York-Presbyterian Hospital, 622 West 168t Street, PH 11- Center, New York, NY 10032 USA

**Keywords:** Total knee arthroplasty, Perioperative pain control, Chronic painful knee arthroplasty, Radiofrequency ablation, Cryoablation, Genicular nerve

## Abstract

**Background:**

Total knee arthroplasty (TKA) is a successful treatment for end-stage osteoarthritis, yet some patients still experience postoperative pain. Genicular nerve radiofrequency ablation (GNRFA) has become a potential modality to address pain in TKA. This systematic review aims to critically analyze the applicability of GNRFA in perioperative pain control prior to TKA, as well as a treatment modality for chronic painful well-appearing TKA.

**Methods:**

PubMed, Medline, EMBASE, Google Scholar, Scopus, and COCHRANE databases, as well as the ClinicalTrials.gov register, were reviewed. The search included randomized controlled trials and cohort studies. The sample population focused on two cohorts; those who underwent TKA and utilized intentional GNRFA as a perioperative pain control modality, and those utilizing the treatment modality for chronic pain in well-appearing TKA. GNRFA was the intervention studied, and postoperative outcomes were compared with the control group, which consisted of those not receiving GNRFA.

**Result:**

Eight total publications were identified as relevant to this search. Among the pre-TKA studies, there was variability in results; these inconsistencies were attributed to a lack of standardization, especially with regard to type, timing, and targeted nerves with ablation. Likewise, while the results were improved among the population with chronic painful TKA receiving GNRFA, these inconsistencies still existed.

**Conclusions:**

Current evidence suggests GNRFA as a possible pre-TKA intervention to potentially minimize opioid consumption, patient-reported pain, length of stay, and increased range of motion and activity. However, the short-lived duration in the setting of chronically painful well-appearing TKA represents a major barrier that warrants further investigation. Limitations include small sample size, heterogeneity, lack of standardization of techniques among studies, and lack of direct comparison and meta-analysis. Further research should focus on the standardization of technique as well as analyzing various patient and health-system-related factors that correlate with sustained positive outcomes.

## Introduction

Total knee arthroplasty (TKA) is among the most common procedures performed nationally, with well-reported success in relieving pain and restoring function in the end-stage arthritic knee [1–3]. While TKA is considered a successful and cost-effective treatment modality, there remains potential for improvement in the satisfaction rates following the procedure [3–5]. As many as 20–30% of patients express a lack of satisfaction with the outcomes following TKA [1], with major cited factors including incomplete pain resolution or restoration of function with continued low mobility or stiffness [2, 3, 6]. While the etiology of dissatisfaction is multifactorial and remains poorly understood, postoperative pain constitutes a commonly cited variable [2, 4, 7, 8].

Pain control in the immediate postoperative setting remains a target for improvement in TKA. When compared with other joint replacement procedures, TKA patients are prescribed a greater total of morphine equivalent volume for a longer period of time, subsequently increasing the risk of opioid dependence [9]. In line with attempts to limit opioid prescription, various modalities for perioperative pain control have been extensively explored, including multimodal pain control and nerve blocks. Commonly used pain control measures include preemptive analgesia, neuraxial anesthesia, peripheral nerve blockades, patient-controlled analgesia, local infiltration analgesia, and oral non-opioid medication [8, 10]. While these modalities contributed to decreasing the reliance on opioids for perioperative pain control, they remain suboptimal with a short duration of impact and with potential risks, such as neuropathy, myositis, and infection [11–13]. Thus, there exists a need for better pain control following TKA. In addition, while TKA is largely successful, a subgroup of patients with well-appearing TKA continue to complain of chronic pain of unidentified etiology with outcomes remaining suboptimal after revision TKA in this population [14].

Genicular nerve radiofrequency ablation (GNRFA) has gained interest as an analgesic modality in TKA. GNRFA was initially used to treat pain associated with osteoarthritis, but now the range of indications has widened to include pain control for perioperative TKA patients and for chronically painful well-appearing TKA [6, 15]. While the topic has been gaining recent traction, a summary of the available literature that highlights the current state of the intervention and potential for improvement remains lacking. This manuscript aims to provide a critical review of the literature and present a comprehensive summary of the current applicability of GNRFA as a pain control modality prior to TKA, as well as a treatment modality for the painful well-appearing TKA.

## Methods

This systematic review of the literature was conducted following the methodology described in the Cochrane Handbook for Systematic Reviews of Interventions. The study is reported following the guidelines of the Preferred Reporting Items for Systematic Reviews and Meta-Analyses statement (PRISMA).

### Eligibility criteria

This review focused on randomized controlled trials (RCT) as well as cohort studies, excluding case reports, case series, surveys, and reviews, and additional inclusion criteria comprised articles published in English, no publication date limit, and full-text availability.

### Literature search

The Population, Intervention, Comparison, and Outcome (PICO) approach was used to guide the search strategy. The sample population focused on two cohorts; those who underwent TKA and utilized intentional GNRFA as a perioperative pain control modality, and those who utilized intentional GNRFA as a treatment modality for chronic pain in well-appearing TKA. These patients were respectively compared with those not receiving GNRFA. The intervention of interest for this study was GNRFA and the dependent variables based on this intervention are postoperative and post-GNRFA outcomes.

The search strategy utilized the PubMed, Medline, EMBASE, Google Scholar, Scopus, and COCHRANE databases, as well as the ClinicalTrials.gov register. The last literature search was performed on 5 October 2022. The RefWorks software was used for referencing. No contact was made with the authors of the articles. Following the identification of articles of interest, the references were manually searched to identify any additional studies meeting the inclusion criteria. The search strategy for each database is listed in Table 1.

**Table 1:** Search strategy per database

Name of database	Search strategy
PubMed	((((radiofrequency ablation) AND (genicular nerve ablation OR neurotomy)) AND (knee arthroplasty))
EMBASE	(‘radiofrequency’/exp OR radiofrequency) AND ablation AND (genicular AND (‘nerve’/exp OR nerve) AND ablation OR ‘neurotomy’/exp OR neurotomy) AND (‘knee’/exp OR knee) AND (‘arthroplasty’/exp OR arthroplasty)
Google Scholar	(((“radiofrequency ablation”) AND (“genicular nerve ablation” OR “neurotomy”)) AND (“knee arthroplasty”)
Cochrane	(((radiofrequency ablation) AND (genicular nerve ablation OR neurotomy)) AND (knee arthroplasty))
SCOPUS	(((“radiofrequency ablation”) AND (“genicular nerve ablation” OR “neurotomy”)) AND (“knee arthroplasty”))

### Screening and assessment of eligibility

After initially screening for duplicates, titles and abstracts were screened. Articles that did not meet the eligibility criteria—language, study design, non-accessible full-text link—as well as those with non-relevant outcomes, were excluded. Outcomes of interest included data on patient mobility assessed via surveys (i.e., PROM, KOOS, SF-12/36) and follow-up evaluations, patient satisfaction assessed with satisfaction surveys, length of hospital stay (LOS) post-TKA, opioid consumption assessed at various postoperative time points based on patient-reported consumption of morphine-equivalent units of opioids, and patient pain levels after the procedure and in follow-up appointments. Pain levels were measured with patient surveys in conjunction with opioid consumption. Adverse events such as intraoperative and postoperative surgical complications were also documented and compared across study groups. LOS was not analyzed among studies using GNRFA as a treatment modality for chronically painful well-appearing TKA. Studies that focus on GNRFA in the setting of osteoarthritis, performance with adjunctive supplementation, and chronic pain not associated with TKA were considered non-relevant outcomes and were excluded.

After the initial screening for eligibility was performed, a screening of full-text articles ensued. Articles that did not have relevant outcomes or focused on a different set of patients (i.e., using genicular nerve ablation to treat osteoarthritis) were further excluded. Two reviewers screened the titles and abstracts to assess for eligibility and final inclusion, with disagreements resolved by consensus and consultation with a senior reviewer.

### Assessment of methodological quality

To assess for quality, the Critical Appraisal Skills Programme (CASP) Checklist was implemented to evaluate each study systematically. This tool provides a way for multiple studies to be measured with the same set of criteria. The questions center around the assessment of clear description and communication of integral parts of a study (validity, results, and application) [16, 17]. For RCTs, the 11-question CASP questionnaire was utilized. For cohort, pilot, and chart review studies, the 14-question CASP questionnaire was utilized. Two questions from this 14-question questionnaire were synthesizing questions to guide the evaluator, rather than objective quality assessment; thus this questionnaire was modified to 12 questions. For both of these studies, a point was awarded if the article included an aspect addressed in a question of the questionnaire, and no points were given if the article did not include said aspect. The points given to each article were then totaled and articles were then ranked on the basis of the sum of points.

The Risk of Bias In Non-randomized Studies—of Interventions (ROBINS-I), a tool generally applied for observational and retrospective studies, was implemented to assess the risk of bias in cohort studies [18]. To assess the bias in the RCTs, a revised version of the questionnaire, RoB 2.0, which analyzes the risk of bias using objective and simple questions, was utilized [19].

### Data extraction

To further extract data from the articles, a literature table was systematically used to collect data from each article. Each article was analyzed for the location, study population, study design, ablation timing relative to GNRFA, the temperature of ablation, target nerves, outcomes, main findings, study limitations, and quality. Outcomes included subjective pain, opioid consumption, function (PROM), length of stay, and adverse events.

## Results

**Fig. 1:**
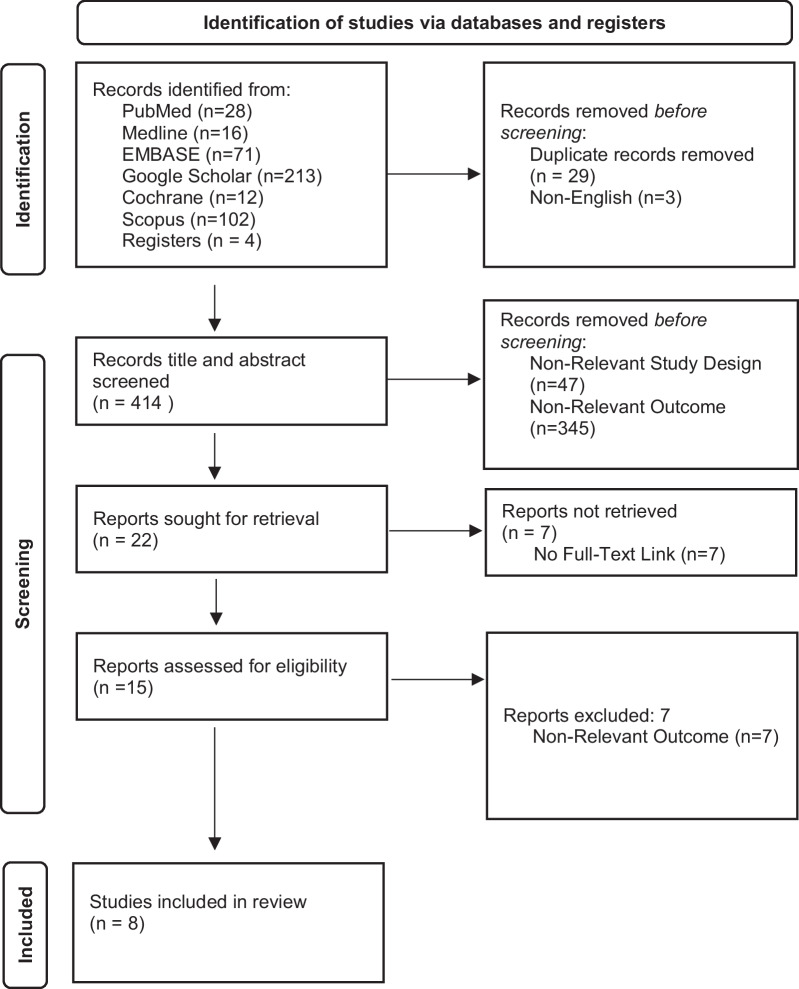
PRISMA flowchart for systematic reviews. *From:* Page MJ, McKenzie JE, Bossuyt PM, Boutron I, Hoffmann TC, Mulrow CD, et al. The PRISMA 2020 statement: an updated guideline for reporting systematic reviews. BMJ 2021;372:n71. https://doi.org/10.1136/bmj.n71. For more information, visit: http://www.prisma-statement.org/

The initial multi-database search returned a total of 446 articles. After excluding articles on the basis of non-relevant study design, non-relevant outcomes, and accessibility, the screening process left 15 articles. The full-text articles were assessed for eligibility, and 7 articles were further excluded on the basis of non-relevant outcomes, thus leaving 8 articles to be included in this review. Figure 1 displays the selection process of eligible articles.

### Study characteristics

While every study analyzed the usage of nerve ablation as a treatment modality, studies differed in the chronological implementation of nerve ablation as a pain control modality around TKA. Study designs varied among the included studies, with four RCTs and four retrospective cohort studies [20–27]. All included studies were published in or after 2016, with patients’ age ranges between 49 years and 85 years. Several studies focused on a smaller subset within this range; Mishra et al., Walega et al., and Dasa et al. focused on an age range between 55 years and 76 years, whereas Khan et al. and Qudsi-Sinclair et al. focused on an age range between 60 years and 80 years [20, 22, 23, 25, 27]. Mihalko et al. studied a population aged 49–79 years, and Erdem et al. focused on 75–85 years of age [24, 26]. The studies in this review included a total of 1082 patients, with 1029 patients in the prior to TKA pain management group and 53 patients in the GNRFA as a treatment modality for the chronically painful TKA group. Among these studies, those not receiving GNRFA or a sham GNRFA—radiofrequency ablation needles placed in proper location without proper neurotomy—acted as the control group; the only exception was in Qudsi-Sinclair et al., in which control was a group receiving an anesthetic and corticosteroid block [22]. Type of GNRFA differed between the studies included in this analysis. Within the pre-TKA group, two studies performed cooled ablation, where temperatures were dropped to −87 °C at the needle tips [24, 27] while the rest performed thermal nerve ablation, where temperatures ranged between 77 °C and 80 °C [20, 21, 23]. Among the painful-TKA group, two studies similarly performed thermal GNRFA [22, 25] while one study performed pulsed radiofrequency ablation with a temperature set to 42 °C [26]. Tables 2 and 3 summarize the characteristics and findings of the included articles.

**Table 2:** What is known about the relationship between genicular nerve ablation administered prior to total knee arthroplasty and the impact on post-surgical outcomes?

Source	Location	Study population	Study design	Thermal or cooled ablation	Ablation timing	Target nerves	Outcome	Main findings	Limitations	Quality assessment
Mishra, 2021	USA—Vanderbilt Orthopedic Clinic	60 patients; aged 55–73 years	Single-center, prospective RCT—sham-controlled and double-blind	Thermal	2–4 weeks prior to TKA	Superior lateral (SL), superior medial (SM), and inferior medial (IM) genicular nerves	Efficacy of conventional GN-RFA for improving postoperative pain at 6 weeks	6-week follow-up: no significant differences between RFA and sham groups	Pain manifestation is not specific and may appear in other aspects of knee—not covered in this study Procedure for GN-RFA was being updated as the study was underway	8/11
Walega, 2019	USA	70 patients; aged 57–75 years	Prospective, randomized, sham-controlled trial	Thermal	2–6 weeks prior to TKA	Superior lateral (SL), superior medial (SM), and inferior medial (IM) genicular nerves,	Opioid consumption, Medication Quantification Scale III (MSQIII) score, and ambulation scores at 48 h, 1 month, 3 months, and 6 months postoperative	No significant difference in opioid consumption, MSQIII scores, and patient mobility at 48-h, 1-month, 3-month, and 6-month mark	Lack of consensus on timing, articular injections, opioid prescribing, and duration of GN-RFA	9/11
Stake, 2022	USA	675 patients	Retrospective cohort	Thermal	96 days prior to TKA	Superior lateral (SL), superior medial (SM), and inferior medial (IM) genicular nerves	2-year surgical outcomes, prolonged opioid use 3–6 months postoperative, 90-day readmission, and medical complications	No differences in the 2-year surgical outcomes Significantly lower prolonged opioid use and TKA-associated complications (blood transfusion, anemia, arrhythmias, and UTIs)	Lack of uniformity in GNRFA procedure and management across sites	12/12
Dasa, 2016	USA—university-based orthopedic practice	100 patients 56–76 years	Retrospective chart review	Cooled	5 days prior to TKA	Anterior femoral cutaneous nerve (AFCN) and infrapatellar branches of the saphenous nerve (ISN)	Hospital length of stay, postoperative opioid requirements, and patient-reported outcomes of pain and function (WOMAC, KOOS, SF-12, and PROMIS)	GNRFA treatment group had a significantly shorter length of stay, significantly greater reductions in patient reported outcomes, and significantly less morphine equivalent narcotics than control group postoperatively	Retrospective, non-randomized, and lack of blinding of patients and investigators Some patients missing data on PROMIS among both study groups	10/12
Mihalko, 2019	USA, single study center	124 patients, aged 49–79 years	Unblinded randomized controlled trial	Cooled	3–7 days prior to TKA	Anterior femoral cutaneous nerve (AFCN) and infrapatellar branches of the saphenous nerve (ISN)	Cumulative opioid consumption at 6 weeks postoperative Pain measures: numerical rating scale (NRS), KOOS, Timed Up and Go (TUG)—measured at 72-h, 2-week, 6-week, and 12-week follow-up assessments	GNRFA significantly reduced opioid consumption at multiple assessments compared with control group Similar percentage of patients among both groups experienced an adverse event	Single site nature of study may not allow for generalizability to larger populations Some patients received general anesthesia; associated with higher rates of infection	9/11

**Table 3:** What is known about the use of genicular nerve ablation as a treatment modality for the painful, uncomplicated TKA?

Source	Location	Study population	Study design	Thermal or cooled ablation	Ablation timing	Target nerves	Outcome	Main findings	Limitations	Quality assessment
Khan, 2022	USA, tertiary academic medical center	19 patients, aged 60–80 years	Longitudinal retrospective pilot study	Thermal	14.6 months after TKA	Suprapatellar nerve (SP), superior lateral (SL), superior medial (SM), and inferior medial (IM) genicular nerves	KOOS questionnaire, visual analog scale (VAS), and the usage of pain medication	After 1 year, KOOS, mean VAS, and WOMAC scores improved significantly among RFA treatment compared with control Less patients used antiinflammatory and opioid medications in the RFA treatment group	Small population size (19 patients) Significantly more female patients than male patients, allowing for selection bias	10/12
Qudsi-Sinclair, 2016	Pain Unit of Hospital Morales Meseguer (Spain)	28 patients, 60–80 years	Double-blind, randomized controlled trial	Thermal	Greater than 6 months after TKA	Superior lateral (SL), superior medial (SM), and inferior medial (IM) genicular nerves	Pain, functionality, quality of life, and patient satisfaction were evaluated using the numerical rating scale (NRS), Oxford Knee score (subjective), Knee Society Score (objective), Short Form Health Survey (SF-36), and Patient Global Impression Scale of Improvement (PGI-I)	There was no significant difference between the treatment groups in the following outcomes: KSS, OKS, PGI-I, SF-36, and NRS No adverse effects associated with GN-RFA were observed—results were similar with analgesic nerve block	Difference in technique could allow for attack at sensory nerves, which may be transmitting pain as well	10/11
Erdem, 2019	Ankara, Turkey	6 patients, aged 75–85 years	Retrospective study	Thermal (42 °C)	2–10 years after TKA	Superior lateral (SL), superior medial (SM), and inferior medial (IM) genicular nerves,	Pain and knee function assessed via VAS and WOMAC at 3 weeks and 3 months Adverse effects were recorded Patient satisfaction was assessed via a 5-point Likert scale	VAS showed a statistically significant reduction from baseline to 3 weeks and 3 months among RFA Significant reduction in baseline WOMAC at 3 weeks, 3 months No adverse effects were recorded Pain did not differ among treatment groups	Small population TKA patients may have had differences in implants, thus ablation of other nerves may have resulted in different outcomes Lack of control group 3 months may be insufficient length of time	10/12

### Study quality assessment

For RCTs, the CASP scores ranged from 8 to 10 out of 11. For the cohort studies, the scores ranged from 10 to 12 out of 12. Overall, these scores indicate proficient quality among these studies; the major setbacks from the RCTs include the inability to apply to the local population [22, 23, 26], inability to provide a greater value to patients [20, 23], whether both treatment and control groups received the same level of care [20, 24], and whether all confounding factors were accounted for [27].

All eight articles were found to have an overall low-to-moderate risk of bias. Among the cohort studies, it was found that these studies had a moderate risk of bias, mostly due to the retrospective nature and lack of randomization among patients and assessors [21, 25–27]. ROBINS-I provides an interpretation of moderate risk of bias as sound evidence yielded by the study, given a nonrandomized design, but is not considered comparable to a well-performed randomized trial. All the RCTs included in this review had low levels of bias, and the main instance of limited bias arose in the form of unblinding [20, 22–24]. Of note, three of the included studies [20, 24, 27] reported authors with industry relations relevant to the GNRFA procedure, with the study by Mihalko et al. receiving direct funding from the industry [24].

### Outcome assessment

#### GNRFA as perioperative pain control prior to TKA

##### Pain and opioid consumption

The reported pain levels differed among the included studies (Table 2). Mishra et al. assessed outcomes using composite pain surveys that analyzed mean pain intensity, ambulation, and Patient Reported Outcomes Measurement Information System (PROMIS) scores and reported no significant difference in any of these measures at the 2-week and 6-week follow-ups.

However, Dasa et al. also demonstrated that, compared with baseline, GNRFA reduced PROMIS pain intensity score significantly at 2 weeks (5.7 to 3.7, *p* < 0.0001) when compared with control (6.4 to 4.8, *p* < 0.176). The authors further noted a significant decrease in PROMIS pain interference scores at the 6-week postoperative mark from baseline in the treatment group (64.1 to 54.1, *p* < 0.0001) but not the control (65.2 to 57.5, *p* = 0.067) [27].

Opioid consumption followed a general trend. Stake et al. noted significantly lower prolonged opioid use, defined as continued opioid use in the 3–6-month postoperative window in the GNRFA cohort (50.81% versus 56.29%, *p* = 0.005). Likewise, Dasa et al. noted a significantly lower cumulative morphine consumption during the 12-week postoperative period among the treatment group when compared with the control (2069.12 ± 132.09 mg versus 3764.42 ± 287.95 mg, *p* < 0.0001) [27]. The authors noted 45% less morphine-equivalent (ME in mg) narcotics consumption in the treatment group. Similarly, Mihalko et al. reported a significant decrease in cumulative opioid consumption at 72 h, 6 weeks, and 12 weeks between GNRFA and control cohorts, but not at the 2-week mark [24]. The authors noted 29% less opioid consumption among the intervention group at the 6-week and 12-week marks [24]. However, Walega et al. reported no significant difference in the consumption of oral opioids at 48 h between the control (144 ME, IQR 112–314, *p* < 0.0001) and treatment groups (192 ME, IQR 105–274, *p* < 0.0001) [20].

##### Outcome and function

Among the pre-TKA articles, three studies noted no difference in outcome measures between GNRFA and control groups [20, 21, 23] (Table 2). Walega et al. reported the longest follow-up for patient-reported outcomes and noted no difference in outcome measures at the 1-, 3-, and 6-month marks, and no significant difference in Medication Quantification Scale III (MSQIII) and Western Ontario and McMaster Universities Osteoarthritis Index (WOMAC) scores. The authors additionally noted no difference between the cohorts with respect to physical functioning, primarily measured by the distance ambulated and the number of stairs climbed [20]. Additionally, the authors noted a significant improvement in Knee Injury and Osteoarthritis Outcome Score (KOOS) scores from baseline (control: 50.8 ± 16.2, treatment: 52.4 ± 16.8) to 6 weeks (control: 55.6 ± 15.3, treatment: 63.8 ± 18.7, *p* < 0.05) and 12 weeks (control: 57.7 ± 16.6, treatment: 69.9 ± 18.0, *p* < 0.05).

The findings of improved outcomes were similarly reported by Mihalko et al., with significant improvement in mean change from baseline of Knee Injury and Osteoarthritis Outcome Score for Joint Replacement (KOOS JR) at the 2-week (treatment: 2.3, control: 1.0, *p* < 0.0001), 6-week (treatment: 9.7, control: 7.7, *p* < 0.0001), and 12-week (treatment: 16.0, control: 14.1, *p* < 0.0001) marks when compared with the standard of care group. Interestingly, the authors reported no significant difference in the Timed Up and Go (TUG) test at any follow-up assessment [24].

##### Length of stay

Dasa et al. noted a significantly shorter length of stay (LOS) among the treatment group (0.8 ± 1.14) in comparison with the control group (1.7 ± 1.01), with a higher proportion of same-day discharge (44.9% versus 14.3%) and discharge at day 1 (49% versus 18.4%) among the treatment group when compared with the control [27]. Otherwise, there was no other significant difference in length of stay among studies (Table 2).

##### Adverse events

Several studies reported on the incidence of adverse events following GNRFA (Table 2). Mihalko et al. reported no significant difference in the incidence of adverse events between the cohorts [24]. In addition, Stake et al. found that there were lower rates of surgery-associated complications, including postoperative anemia, atrial fibrillation, arrhythmia, blood transfusion requirement, and urinary tract infection among the GNRFA when compared with the non-GNRFA group [21]. While not an adverse event, Walega et al. noted no significant difference in the incidence of postoperative analgesia effects (sedation, nausea, delirium, pruritus) between the control (29.6%) and the experimental arm (24.2%), *p* = 0.016 [20].

#### GNRFA as a treatment for chronically painful in well-appearing TKA

##### Pain and opioid consumption

Findings among these studies are highlighted in Table 3. Erdem et al. noted a 50% improvement in VAS pain score at the 3-week and 3-month marks among 67% of patients in the intervention group. The authors additionally reported a significant improvement in WOMAC when comparing baseline (65.1 ± 2.8), 3-week (40.7 ± 3.2, *p* < 0.01), and 3-month (46.2 ± 4.0, *p* < 0.01) scores in the intervention group. Qudsi-Sinclair et al. did not compare their results with a control group, but rather to another experimental measure and noted a numeric rating scale (NRS) value of 7.07 before treatment that decreased to 4.47 at the 6-month mark, and 4.93 at the 1-year mark following GNRFA. In addition, the authors also reported a significant improvement in Oxford Knee score (OKS) (9.62 ± 9.45, *p* < 0.01) and Knee Society Score (KSS) (17.62 ± 13.11, *p* < 0.01) after 1 year [22].

Among the chronically painful TKA, Khan et al. also noted a lower consumption of antiinflammatories and opioids in the GNRFA group compared with the control [25]. Qudsi-Sinclair et al. found that 7 of the 14 GNRFA patients needed oral opioid treatment before infiltration, and this number dropped to 3 and 2 after 6 months and 12 months, respectively [22].

##### Outcome and function

When analyzing the outcomes of GNRFA as a treatment for the chronically painful well-appearing TKA, the results among the studies were largely similar (Table 3). Khan et al., in a cohort averaging 14.6 months postoperatively from primary TKA, reported a significant improvement in patient-reported outcomes for more than 1 year following GNRFA [25]. The authors noted improvement in all categories within the mean KOOS (from 35.0 to 64.2, *p* < 0.0001), mean visual analog scale (VAS) (from 8.30 to 2.45, *p* < 0.0001), and mean WOMAC scores (from 36.9 to 62.0, *p* < 0.0001) in the intervention groups when compared with the control.

##### Adverse events

Qudsi-Sinclair et al. reported no adverse events but noted some patients felt specific pain when the GNRFA cannula touched the periosteum [22]. Otherwise, no adverse events were reported among these studies.

## Discussion

GNRFA has been gaining recent focus as an attractive pain control modality perioperatively in the setting of TKA and for the well-appearing uncomplicated chronically painful TKA. In this systematic review, we note discrepancies in the literature among the several primary outcomes, when GNRFA was utilized as a perioperative pain control modality prior to TKA. When analyzing pain and opioid consumption as primary outcomes, two studies reported no significant difference with GNRFA over time periods varying from 48 h to 6 months [20, 23]. However, three studies found that GNRFA directly impacted immediate and prolonged opioid usage, and an improvement in postoperative pain scores ranging from 72 h to 1 year [21, 24, 27]. These same studies reported a significant decrease in LOS, improvement in ambulation, and a significant impact on adverse outcomes associated with surgery [21, 24, 27]. With regard to the application as a treatment to the chronically painful well-appearing TKA, the studies noted an improvement in pain scores and ambulation, ranging from 3 weeks to 1 year in continued improvements [22, 25, 26], with two studies reporting a reduction in opioid consumption [22, 25].

### Pre-TKA

GNRFA has been used consistently in the past to address knee pain associated with osteoarthritis to delay the need for TKA [20, 23, 24]. This indication has been recently expanded to include perioperative pain control in TKA. As a result, the primary outcomes and measures differed across the articles and no consistent reporting was noted in the included studies. Collectively, these studies demonstrate that GNRFA is likely successful in decreasing pain and postoperative opioid use, improving postoperative knee functionality, and decreasing surgical complications. While the contribution to pain control, and potentially subsequent improved function, could be easily explained, the association with lower complication rate is less clear and remains less understood. It is important to note that among these outcomes, several gaps and discrepancies were identified.

The major discrepancy noted in the literature is the lack of a universal or standardized approach to GNRFA, especially with regard to timing and type of ablation. Walega and Mishra performed thermal ablation 2–6 weeks prior to TKA, and these studies showed no difference in effect between the GNRFA group and the control group [20, 23]. Walega et al. found no difference in opioid consumption, patient-reported measures, or functionality between treatment and control groups at 48 h. However, Mihalko and Dasa performed cooled ablation 3–7 days prior to TKA, and these studies displayed marked improvements in LOS, total opioid consumption, and patient-reported pain and ambulation [24, 27]. Stake et al. stated that the procedure was done on average 96 days prior to TKA, however, the standard deviation was 91 days [21]. Given these results and the notable differences in reported outcomes, it could be stated that the lack of impact with GNRFA might be related to type and timing, and it could be extrapolated that optimally cooled ablation should be performed within 1 week prior to TKA. These findings could be explained by previous studies reporting the impact of GNRFA within 2 weeks of the intervention [20]. While the available evidence points toward the highest benefit of GNRFA within 7 days of primary TKA, further studies are needed to investigate the optimal timing for GNRFA to allow for the greatest therapeutic effect on pre-TKA patients [20, 23].

An additional discrepancy exists with regard to specific targeted nerves of ablation. In the studies by Mishra et al. and Walega et al., the authors targeted the superior lateral (SL), superior medial (SM), and inferior medial (IM) genicular nerves, all derived from the sciatic nerve, and these targets resulted in no significant difference in outcomes from the control group [20, 23]. Furthermore, Mihalko et al. and Dasa et al. targeted the superficial genicular nerves arising from branches of the anterior femoral cutaneous nerve (AFCN) and infrapatellar branches of the saphenous nerve (ISN) on the anterior aspect of the knee, and these studies reported significant improvement in outcomes among the intervention group compared with the control [24, 27]. Similarly, other studies have suggested up to ten ablation sites instead of the standard 2–4 ablation sites [23, 28]. While further studies are needed to assess the optimal sites for ablation, it appears that the anterior genicular branches arising from the AFCN and ISN should be targeted with the procedure [20, 23, 24, 27].

This overall lack of a uniform procedure manifests as the main disadvantage within the available evidence for GNRFA. A discrepancy in timing and target sensory nerves for ablation has led to inconsistent results in the literature. Furthermore, pain may manifest in the inferolateral or posterior compartment of the knee, which differs from the target sites of ablation, thus the incidence of this specific localization of pain should be explored in future research [23].

In a retrospective cohort analysis, Stake noted that GNRFA recipients were more likely to have more comorbidities yet displayed lower rates of surgical complications such as blood transfusions, anemia, arrhythmias, and urinary tract infections [21]. Similar findings were noted in the studies by Dasa and Mihalko [24, 27]. In an attempt to explain such findings, Stake reports GNRFA commonly performed at tertiary-care centers with adequate equipment and a greater ability to medically optimize patients perioperatively and subsequently decrease risk of complication [21]. While such an explanation could be viable, future research must explore alternative factors leading to an improved rate of complications among GNRFA recipients. In the TKA setting, the evidence in favor of GNRFA as a successful perioperative pain control modality remains weak, however, current data point toward the potential for improvement in patient-reported outcomes and pain levels, decreased opioid consumption, length of stay, and infection risk.

### Chronic painful TKA

In the setting of the chronically painful well-appearing uncomplicated TKA, the three studies assessing the impact of GNRFA reported significant improvement in pain, patient-reported outcomes, and overall functionality [22, 25, 26]. Specifically, Erdem et al., using pulsed radiofrequency, noted improvement from baseline through 3 months, and Khan et al. noted significant improvement through 12 months. While Qudsi-Sinclair et al. noted a significant improvement in pain up to 6 months, the authors note pain returned to baseline after 12 months [22]. Of note, in this study, the authors note a population composed solely of elderly patients aged 60–80 years, thus posing a challenge to measuring opioid consumption given required analgesics for non-TKA-related conditions. Khan et al. measured opioid consumption within a similar population and found that while GNRFA can serve as an alternative treatment option, there is still a need for future research among a larger population [25].

Much like pre-TKA treatment, one of the main weaknesses in evidence for GNRFA as a treatment modality for painful TKA is the inconsistency of the procedure. Khan et al. focused on SL, SM, IM, and suprapatellar genicular nerve (SP) nerves, whereas the other studies targeted the SL, SM, and IM nerves [22, 25, 26]. Furthermore, among the three studies in this subset, none performed cooled GNRFA; Qudsi-Sinclair et al. and Khan et al. performed neurolysis at 80 °C, while Erdem et al. performed ablation at 42 °C [22, 25, 26]. Overall, it could be concluded that GNRFA is a successful modality in improving symptoms in the painful TKA and could be considered in patients with unclear etiology for pain or who are unable to undergo revision TKA. Even among TKA determined to have a good outcome, nearly 20% of patients experience pain afterward [14]. In this population with well-appearing components, there is ample evidence in the literature on poor outcomes following revision surgery based solely on chronic pain [14, 25]. This unexplained pain is often reported as neuropathic pain, thus posing the need for an alternative modality such as GNRFA [25, 29]. While the available evidence supports the potential use of GNRFA, there is a lack of studies assessing the impact of cooled GNRFA among the well-appearing and chronically painful TKA.

While radiofrequency ablation has positives, there are some patient factors that can contribute to its effectiveness. As Erdem et al. elucidate, post-surgical changes from TKA can pose difficulty in locating the targets for GNRFA [26]. However, if successful in locating targets, GNRFA can be useful as a treatment modality for the uncomplicated well-appearing painful TKA [25]. Future research should explore whether these targets are adequate and should be done with an adequate sample size to be generalizable to the population, as all three studies had small sample sizes.

### Limitations

There exist several limitations regarding this study. The inclusion criteria led to a limited sample size, thus preventing a meta-analysis from being performed. Likewise, a lack of direct comparison among studies was also identified as a limitation of this study. Each paper included a unique sample and study procedure, thus the heterogeneity of GNRFA techniques among included studies is also a limitation of this paper. Finally, outcome measurements were not standardized across each study, thus proving to be a limitation of this review.

### Conclusions

As an effective mode of pain control in the management of osteoarthritis and chronic knee pain, GNRFA is a potentially beneficial treatment modality for pain associated with TKA. GNRFA can demonstrate the possibility of minimizing opioid consumption, patient-reported pain, length of stay, and increasing range of motion and activity. However, the standardization of ablation sites and timing remains a major barrier to systemic implementation, and the short-lived duration in the setting of chronically painful well well-appearing TKA represents a major barrier that warrants further assessment. As such, future studies with standardized timing for intervention and target nerves, as well as assessing various patient and health-system-related factors that correlate with a sustained positive outcome, are needed.

## Data Availability

Not applicable.
